# Elucidation of mechanisms underlying active oxygen burst in *Citrus sinensis* after *Diaporthe citri* infection using transcriptome analysis

**DOI:** 10.3389/fmicb.2024.1425441

**Published:** 2024-08-29

**Authors:** Tiantian Liu, Zehua Zhou, Changwei Luo, Hua Luo, Jun Tang, Xiaojiang Shi, Diping Li, Qiong Zhang, Jin Li, Yonggang Xia, Na Song, Tuyong Yi

**Affiliations:** ^1^Hunan Provincial Key Laboratory of Plant Diseases and Pests, College of Plant Protection, Hunan Agricultural University, Changsha, Hunan, China; ^2^Shaoyang Academy of Agricultural Sciences, Shaoyang, Hunan, China; ^3^Human Academy of Forestry, Changsha, Hunan, China

**Keywords:** citrus melanose, *Diaporthe citri*, RNA-Seq, sweet orange, reactive oxygen species

## Abstract

**Introduction:**

Reactive oxygen species (ROS) generation is a common disease defense mechanism in plants. However, it is unclear whether *Citrus* host activates defense response against *Diaporthe citri* causing citrus melanose disease by producing ROS, and the underlying molecular mechanisms are unknown.

**Methods:**

DAB staining and RNA-Seq technology were used to compare the active oxygen burst and differential gene expression, respectively, in uninfected and infected *Citrus sinensis* leaves at different time points during *D. citri* infection *in vivo*. The functions of *CsRBOH* (a significant DEG) were confirmed in *N. benthamiana* through the *Agrobacterium*-mediated transient expression system.

**Results:**

DAB staining indicated that *C. sinensis* initiated defense against *D. citri* infection within 24 h by generating ROS. Illumina sequencing revealed 25,557 expressed genes of *C. sinensis*. The most upregulated DEGs (*n* = 1,570) were identified 72 h after fungal inoculation (sample denoted as CD72). In the CD72 vs. Cs (samples at 0 h after fungal inoculation) comparison, the KEGG pathway category with the highest number of genes (*n* = 62) and most significant enrichment was Protein processing in endoplasmic reticulum, followed by Glutathione metabolism and MAPK signaling pathway-plant. GO analysis revealed that the DEGs of CD72 vs. Cs related to active oxygen burst and chitin recognition were significantly grouped into the regulation of biological processes and molecular functions, with GO terms including response to ROS, response to fungus, and oxidoreductase activity. Remarkably, *CsRBOH* was significantly enriched in the GO and KEGG analyses, and its expression pattern in qRT-PCR and DAB staining results were consistent. Among the 63 ROS-related DEGs, HSP genes and genes associated with the peroxidase family were highly significant as revealed by protein–protein interaction networks. Furthermore, ROS accumulation, cell death, and upregulation of defense-related genes were observed in *N. benthamiana* leaves with *CsRBOH* expressed through the *Agrobacterium*-mediated transient expression system.

**Conclusion:**

Our findings suggested that *C. sinensis* activates *CsRBOH* and ROS-related genes, leading to ROS accumulation to resist the invasion by *D. citri*. This study laid the foundation for future research on molecular mechanisms and breeding of *C. sinensis* cultivars resistant to citrus melanose.

## 1 Introduction

*Citrus* (family Rutaceae), with several important species including oranges, mandarins, and grapefruits, has significant economic value globally. In China, the cultivated area of *Citrus* is 2854.16 thousand hm^2^, with production accounting for approximately 1/3^rd^ of that in the world (Deng, 2022). *Citrus* fruits and flavors possess a diverse range of applications. *Citrus* fruits provide essential vitamins, antioxidants, minerals, and dietary fiber that are crucial for maintaining overall nutritional well-being (Ran, 2022). *Diaporthe citri* (order Ascomycota) is a weak fungal parasite causing citrus melanose (CM) disease in various economically important *Citrus* crops (Chaisiri et al., 2022). After the 1990s, CM became a significant issue in *Citrus* cultivation with the spread of the pathogen in several areas globally (Swingle and Webber, 1896). *Diaporthe citri* has been reported in various *Citrus* hosts and related species globally, and *D. citri*–host combinations are listed by the USDA’s Agricultural Research Service’s Systematic Mycology and Microbiology Laboratory and the Centre for Agriculture and Bioscience International (Chaisiri et al., 2020). *Diaporthe citri* can thrive in various climates, including temperate, tropical, and subtropical regions (Fang, 2017).

*Citrus* trees with *D. citri* infection exhibit various symptoms such as melanose, twig blight, gummosis, and stem-end rot (Mondal et al., 2007; Huang et al., 2013; Udayanga et al., 2014; Guarnaccia and Crous, 2017). The anamorph (imperfect stage) of *D. citri* is *Phomopsis citri* (Fawcett, 1912). Invasion of *Citrus* leaves and fruits by *D. citri* usually occurs via the formation of conidiospore of *P. citri* and ascospore of *D. citri* over the leaf and fruit surface. They penetrate the host tissues through epidermal and cortical cells, and finally, CM symptoms appear in the host plant (Kuhara, 1999). Chemical or physical approaches are not completely effective in controlling this disease, and the methods to reduce the load of pathogen are time-consuming and laborious. For example, Xu et al. (2019) proposed spraying 12% carbendazim +63% Mancozeb and 7.5% tetraconazole +9.5% Azoxystrobin 5 times a year to control *D. citri*. In addition, *D. citri* grows rapidly in dry environments within a temperature range of 17 to 35°C, and its infection cycle is up to 6 months (Mondal et al., 2007).

*D. citri* infects most citrus plants such as sweet oranges and grapefruit, whose plantation should be avoided in areas with high rainfall (Barkley et al., 2014; Chaisiri et al., 2020). According to Mondal et al. (2004), all citrus varieties are susceptible to CM disease, particularly lemon and grapefruit. Zhou et al. (2015) characterized the resistance of 73 germplasm resources of *Citrus* spp. to CM disease via inoculating individual leaves and fruits with *D. citri*. They reported that all 73 germplasm resources were not immune to CM disease; however, most pomelo and lemon species exhibited high resistance. Pink Thompson grapefruit, some broad-skinned and hybrid mandarins, and eight *C. sinensis* (L.) Osbeck germplasms were susceptible to *D. citri* infection.

When the spores of *D. citri* land on young citrus leaves, they can germinate and infect the epidermal tissues of leaves under suitable ambient temperature and humidity conditions (Li, 2022). The epidermal cells of leaves infected with *D. citri* mycelium turn brown and necrotic, and the surrounding cells abnormally divide, proliferate, protrude, and expand under the influence of gamma-amino-n-butyric acid (an inducer of cell division). This process eventually leads to the formation of healing tissues (callus) consisting of 10–12 layers of cells. In severe cases, a sandy skin symptom may appear on the surface of branches and fruits as a result of this defense mechanism against *D. citri* infection. This barrier, composed of necrotic cells, healing tissues, and periphyton, separates healthy citrus tissue from pathogens while protecting the normal physiological activities of citrus tissues (Arimoto et al., 1982; Gopal et al., 2014; Fazalur, 2020; Huang et al., 2024). Black punctate spots caused by D. citri infection do not exhibit spore-producing structures such as ascospores; hence, they cannot serve as a source for reinfection in black spot disease in fields. Therefore, it is believed that “black spot” symptoms are an outcome of citrus host’s defense response against further infection by *D. citri* (Jiang et al., 2012).

Reactive oxygen species (ROS), including superoxide anion radical (O^2−^) and hydrogen peroxide (H_2_O_2_), play a crucial role in multiple signal transduction processes in plants (Mittler et al., 2011; Hernandez et al., 2019; Zhao et al., 2020). ROS signaling plays a key role in plants’ defense against pathogen invasion (Barba-Espín et al., 2021; Király et al., 2021). In the plant-pathogen interaction system, ROS are primarily generated through the mediation of NADPH oxidases (Torres and Dangl, 2005). However, it remains unclear whether the *Citrus* host activates the defense response against *D. citri* by producing ROS, and the underlying molecular mechanisms are unknown.

In recent years, progress in the elucidation of the *Citrus* genome and annotation has promoted high-throughput sequencing (HTS) studies, which aimed to gain a comprehensive understanding of the molecular mechanisms underlying the host response to pathogenic infection (Xu et al., 2013). In the research on Citrus, HTS analysis of mRNA is extensively used for elucidating the host’s response to pathogens such as *Candidatus Liberibacter asiaticus* (*C*Las; Martinelli et al., 2012; Fu et al., 2016; Wang et al., 2016; Arce-Leal et al., 2020) and *Citrus tristeza virus* (Fu et al., 2016; Visser et al., 2017). However, transcriptomic studies on *D. citri* infection are scarce. It is necessary to obtain detailed insights into the pathogenesis of *D. citri* via extensive transcriptome analysis during its interaction with the host.

In this study, we aimed to compare the transcriptome of uninfected and infected *C. sinensis* leaves at various stages of *D. citri* infection *in vivo* using RNA-Seq technology. The functional annotation of the differentially expressed genes (DEGs) was performed using Gene Ontology (GO) and Kyoto Encyclopedia of Genes and Genomes (KEGG) functional enrichment analyses, and potential genes of *C. sinensis* associated with *D. citri*-induced active oxygen burst were identified. This study provided significant insights into the molecular mechanisms contributing to CM disease development in natural environments.

## 2 Materials and methods

### 2.1 Plant material, fungal strain, and culture conditions

One-year-old sweet orange cultivar plants are known to be susceptible to *D. citri* (Deng, 2022) were provided by the National Center for *Citrus* Improvement (Changsha) of Hunan Agricultural University. They were cultivated in a partially regulated greenhouse environment at approximately 30°C with 60% relative humidity. The highly pathogenic *D. citri* HJG-1 fungal strain, isolated by our research group, was cultured on potato dextrose agar (20% potato, 2% glucose, and 2% agar) at 28°C.

For spraying on the *C. sinensis* leaves to initiate infection, the conidia of *D. citri* were collected after culturing *D. citri* on OMA (3% oatmeal and 1% agar in 1 L of distilled water) agar medium for 20 days at 28°C under 12 h photoperiod.

### 2.2 Inoculation of *Diaporthe citri* on the leaves and disease progression assay

To understand the epidemiology of the disease at different stages, disease progression assay was performed. First, fresh leaves and branches of *C. sinensis* were surface sterilized using 70% ethanol. After air drying, 10 mL conidial suspension (1.0 × 10^5^ conidia mL^−1^ in sterile water) was directly sprayed on the leaf blade surface of plants 20 days after leaf spreading. After this, the leaves were sealed in a plastic bag, and a sponge soaked with sterile water was placed in the bag for 24 h to provide humid environment (Liu et al., 2018). The experiment involved three replicate plants with inoculation performed on each shoot containing at least five leaves. The leaves not sprayed with the conidia suspension were used as the control.

To observe the germination and infection process of *D. citri* in young leaves, fluorescent staining was used to stain *D. citri* cells on the leaves. The staining method was a modification of a technique described by Liu et al. (2016) to observe infections in *Phytophthora infestans* using the solophenyl flavine dyes. After 0, 12, and 24 h of inoculation with *D. citri*, the leaves were immersed in 0.00005% (M/V) direct yellow 96 (0.1 M solution in Tris–HCl buffer at pH 8.5) for 5 min. The samples of leaves after inoculation for 48, 72, and 120 h were decolorized using glacial acetic acid: 95% ethanol (1:3; V/V). Then, the samples after discoloration are dyed up. The stained *D. citri* cells were observed using epifluorescence microscopy with a Zeiss Axio Vert A1 (40×) microscope at excitation wavelength of 365 nm.

The oxidative response of host to pathogenic infection was observed using 3,3-diaminobenzidine (DAB) staining (Liu et al., 2020; Wang, 2023). H_2_O_2_ is the main component of ROS burst in plants. DAB is oxidized by H_2_O_2_ to generate dark brown spots. At various time points (12, 24, 48, 72, and 120 h after inoculation with *D. citri*), the leaves were immersed in DAB buffer (pH = 3.8; Coolaber, Beijing, SL1805). The samples were incubated in dark at room temperature for 8 h. Next, the samples were bleached via heating in ethanol: glacial acetic acid (3:1) for 15 min (Zha, 2022). The leaves were photographed to compare the dark brown spots. In the control, water was inoculated instead of *D. citri*. Inoculation of sterile water as a control. This experiment was performed in triplicates.

### 2.3 Transcriptome sequencing

The seedling leaves were collected at specific time intervals (0, 12, 24, 48, 72, and 120 h; samples denoted as Cs, CD12, CD24, CD48, CD72, and CD120, respectively) after inoculation for RNA-Seq, with the control group containing samples collected at 0 h. From three plants, five leaves were obtained from each at every time point. Samples from each treatment were individually collected, rapidly frozen in liquid nitrogen, and stored at −80°C for subsequent RNA extraction for transcriptome sequencing.

RNA was extracted using TRIzol® Reagent (TransGen Biotech) as per the manufacturer’s instructions. Subsequently, the quality of the extracted RNA was assessed using 5,300 Bioanalyser (Agilent). The RNA was quantified using ND-2000 (NanoDrop Technologies). Only high-quality RNA samples (OD260/280 = 1.8–2.2, OD260/230 ≥ 2.0, RIN ≥ 6.5, 28S:18S ≥ 1.0, and amount > 1 μg) were used to construct the RNA-Seq transcriptome library using 1 μg of RNA sample and Illumina® Stranded mRNA Prep kit (Illumina, San Diego, CA). The processes such as RNA purification, reverse transcription, library construction, and sequencing were conducted by Shanghai Majorbio Bio-pharm Biotechnology Co., Ltd. (Shanghai, China) as per the instructions by Illumina (San Diego, CA).

### 2.4 Data processing and analysis of DEGs

The paired-end reads were subjected to trimming and quality control using fastp (Chen et al., 2018) with default settings. The clean reads were separately aligned to the reference genome (*Citrus sinensis* v3.0: http://citrus.hzau.edu.cn/download.php; Liu et al., 2022) in orientation mode using HISAT2 software (Kim et al., 2015). The assembled mapped reads of each sample were generated using StringTie (Pertea et al., 2015) through a reference-based approach. To identify the DEGs between two distinct samples, the transcript levels were determined using the transcripts per million reads (TPM) method. Gene abundance was quantified using RSEM (Li and Dewey, 2011), followed by differential expression analysis using DESeq2 software (Love et al., 2014). Genes exhibiting |log_2_fold change (FC)| ≥ 1 and false discovery rate (FDR) < 0.05 as determined by DESeq2 were considered as significantly differentially expressed.

In addition, GO and KEGG functional enrichment analyses were performed using Goatools (Klopfenstein et al., 2018) and Python scipy,^1^ respectively, to identify the DEGs exhibiting significant enrichment in GO terms and metabolic pathways compared to the entire transcriptome. The Bonferroni-corrected *p*-value threshold was set at <0.05 for this analysis.

Protein–protein interaction (PPI) networks were studied using STRING database and Cytoscape software.

### 2.5 Transient expression of *CsRBOH* in *Nicotiana benthamiana*

The open reading frame of *CsRBOH* was amplified using PCR and inserted into the binary vector pCAMBIA-1300 via homologous recombination. The recombinant constructs were transformed into *A. tumefaciens* strain GV3101-P19 via electroporation, followed by transient expression in 6-week-old *N. benthamiana* leaves using the methods described previously (Ma et al., 2012; Zhang et al., 2021). For the *A. tumefaciens*-mediated transient expression assay, Bcl2-associated X protein (BAX) and the binary vector pCAMBIA-1300 were used as the positive and negative controls, respectively. Each treatment was conducted on three leaves obtained from three separate plants, and the experiment was repeated a minimum of three times. DAB staining was used to detect ROS accumulation in *N. benthamiana* leaves 2–3 days postinfiltration (dpi; Ma et al., 2012). Trypan blue staining was performed to detect cell death in *N. benthamiana* leaves 7–8 dpi (Ma et al., 2012).

### 2.6 qRT-PCR validation of DEGs and gene expression analysis

Total RNA was extracted from 0.1 g of *C. sinensis* leaves collected at various time points after *D. citri* infection and from 0.1 g of *N. benthamiana* leaves 1, 3, and 7 dpi using TRIzol® Reagent as per the manufacturer’s instructions. Subsequently, 1 μg of total RNA was reverse transcribed using HiScript II Q RT SuperMix (Vazyme) and subjected to qPCR (+gDNA wiper).

In total, 14 DEGs in *C. sinensis* were randomly selected to validate the results of RNA-Seq. The *COX* gene served as an internal control. Four defense-related genes in *N. benthamiana* were selected for expression analysis using qPCR after introducing *CsRBOH*. *NbActin* served as an internal control (Sun et al., 2022). Samples of *N. benthamiana* with pCAMBIA-1300 were set as the control. Sequences of primers are given in Supplementary Table S1 (Zhang et al., 2015; Song et al., 2021; Chen et al., 2022; Qin, 2023). The experiments were conducted using a Real-Time PCR Detection System (Bio-Rad) and ChamQ Universal SYBR qPCR Master Mix, as per the manufacturer’s instructions (Vazyme). Relative gene expression was calculated as described previously (Francis et al., 2009). Each sample was analyzed in triplicates, and the entire procedure was repeated three times.

### 2.7 Statistical analysis

Statistical analysis was conducted using the DPS v15.10 software. Significant differences were assessed using Duncan’s multiple range tests at a significance level of *p* < 0.05. The findings were reported as the mean ± standard deviation (SD) of three biological replicates.

## 3 Results

### 3.1 *Diaporthe citri* infection induces active oxygen burst response in *Citrus sinensis*

**Figure 1:**
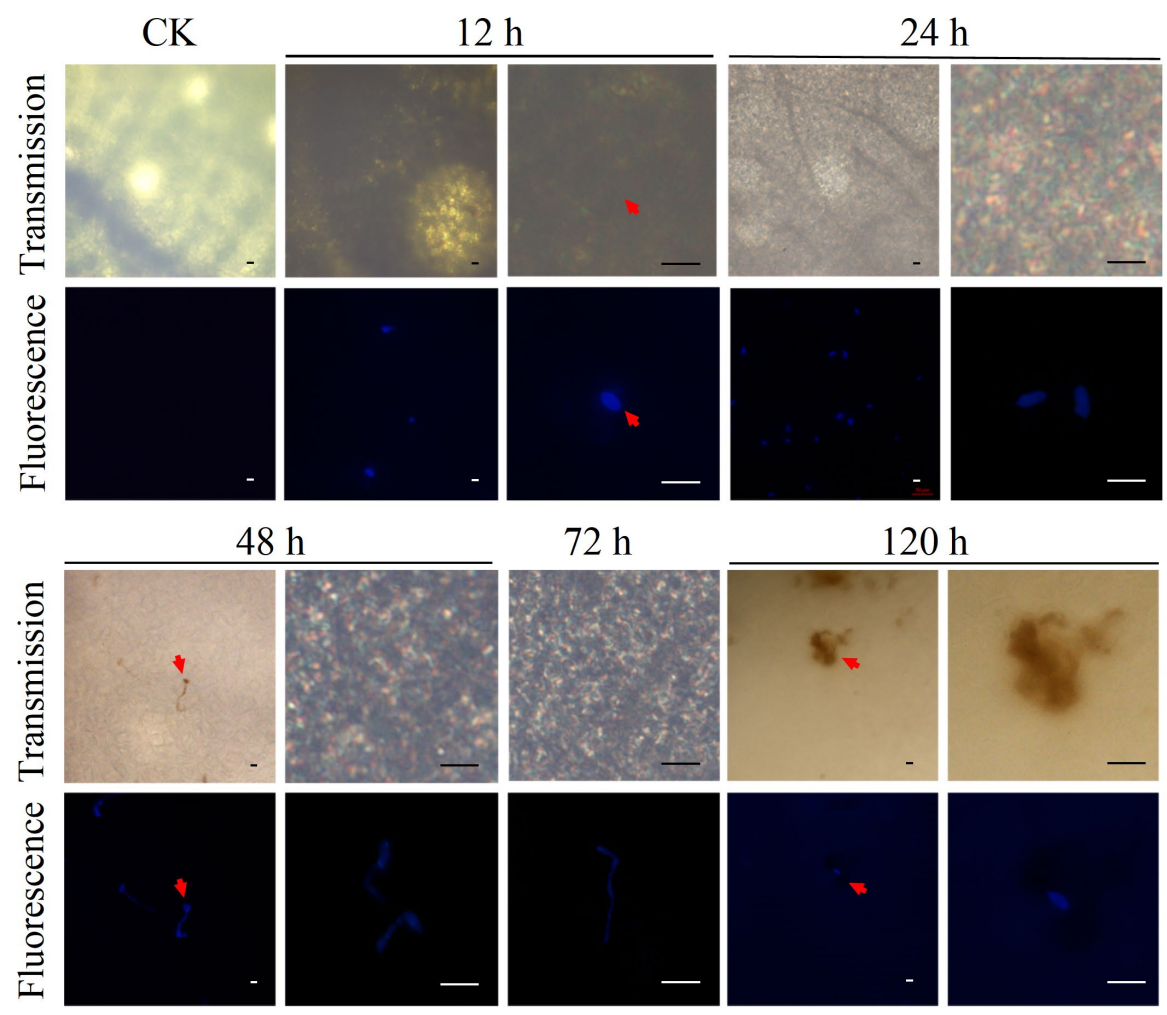
Fluorescence staining of *D. citri* cells was conducted to investigate the dynamics of *D. citri* infection in *C. sinensis* plants. Microscopic analysis of 20-day-old shoot of *C. sinensis* inoculated with the conidial suspension of *D. citri* HJG-1 (1 × 10^5^ spores/mL) and stained using direct yellow 96. The spore germination and infection were observed at 12, 24, 48, 72, and 120 h after inoculation. Each panel shows transmission light (Transmission) and fluorescence microscopic results with excitation wavelength of 380 nm. Scale bar = 20 μm.

**Figure 2:**
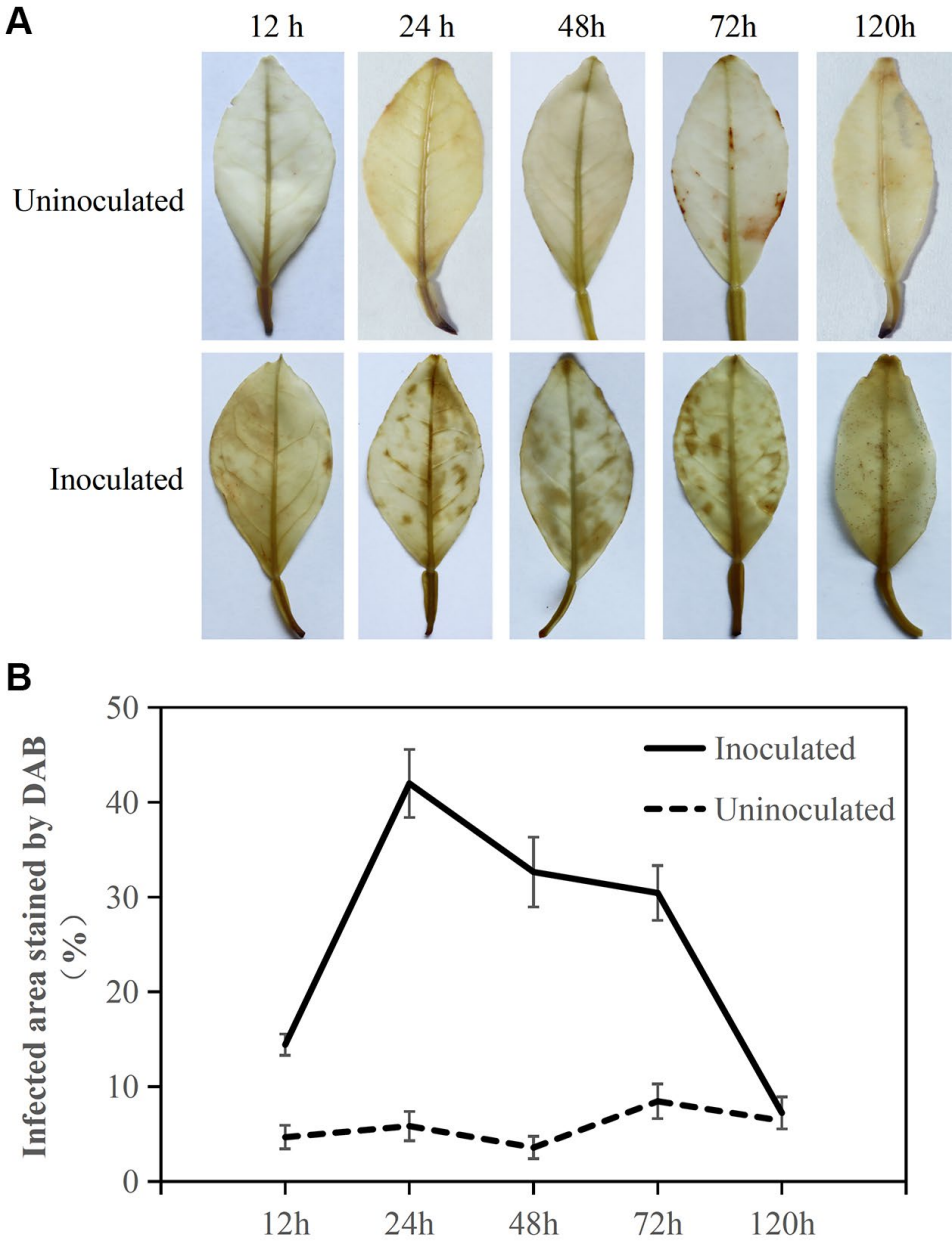
DAB staining to determine active oxygen burst response in *Citrus sinensis* after *Diaporthe citri* infection. **(A)** Sequential images depicting brown spots corresponding to ROS accumulation during the infection of *C. sinensis* leaves by *D. citri*; **(B)** The percentage (%) of infected leaf area stained brown after DAB staining relative to the total area of inoculated leaves, with calculations performed on five infected *C. sinensis* leaves. Inoculation of sterile water as a control. The experiment was repeated three times, and consistent results were obtained. Error bars represent standard deviation.

Fluorescence staining of *D. citri* cells and DAB staining were used to investigate the dynamics of *D. citri* infection in *C. sinensis* plants (Figures 1, 2). The *D. citri*-*C. sinensis* interaction was studied at the microscopic level by fluorescent staining of *D. citri* cells on the leaves (Figure 1). Infection process was observed at 12, 24, 48, 72, and 120 h after inoculation. After 12 h, the spores were found attached to the leaves; they had absorbed water and were swollen as seen before germination. At 24 h, the spore germination was observed on the leaves. From 48 to 72 h, the mycelium grew and gradually infected the leaves. However, at 120 h, infection progression was suppressed. At the same time, consistent with the symptoms observed by naked eye, necrotic spots appeared on the host at 120 h, suggesting that the infection process was complete (Figure 1; Supplementary Figure S1). Therefore, the appearance of symptoms of CM disease was completed by approximately 120 h in *C. sinensis* leaves.

DAB staining was used to estimate ROS accumulation in the leaves in response to *D. citri* infection (Figure 2). *Citrus sinensis* infected with *D. citri* exhibited reddish-brown spots on the surface of the inoculated blade at 12, 24, 48, 72, and 120 h. The proportion of leaf area stained with brown spots was nearly 20% at 12 h. Over 30% of the leaf area was stained brown at 24, 48, and 72 h. At 120 h, the stained leaf area proportion reduced to approximately 10%, still exhibiting the presence of ROS. In contrast, ROS accumulation was barely detectable in the leaves inoculated with sterile water. These findings suggested that within 24 h, the presence of ROS in *C. sinensis* can hinder *D. citri* infection, and it is crucial for *D. citri* to eliminate ROS in the host to facilitate its subsequent growth during the infection process.

### 3.2 Transcriptome analysis of *Citrus sinensis* leaves after *Diaporthe citri* infection

**Figure 3:**
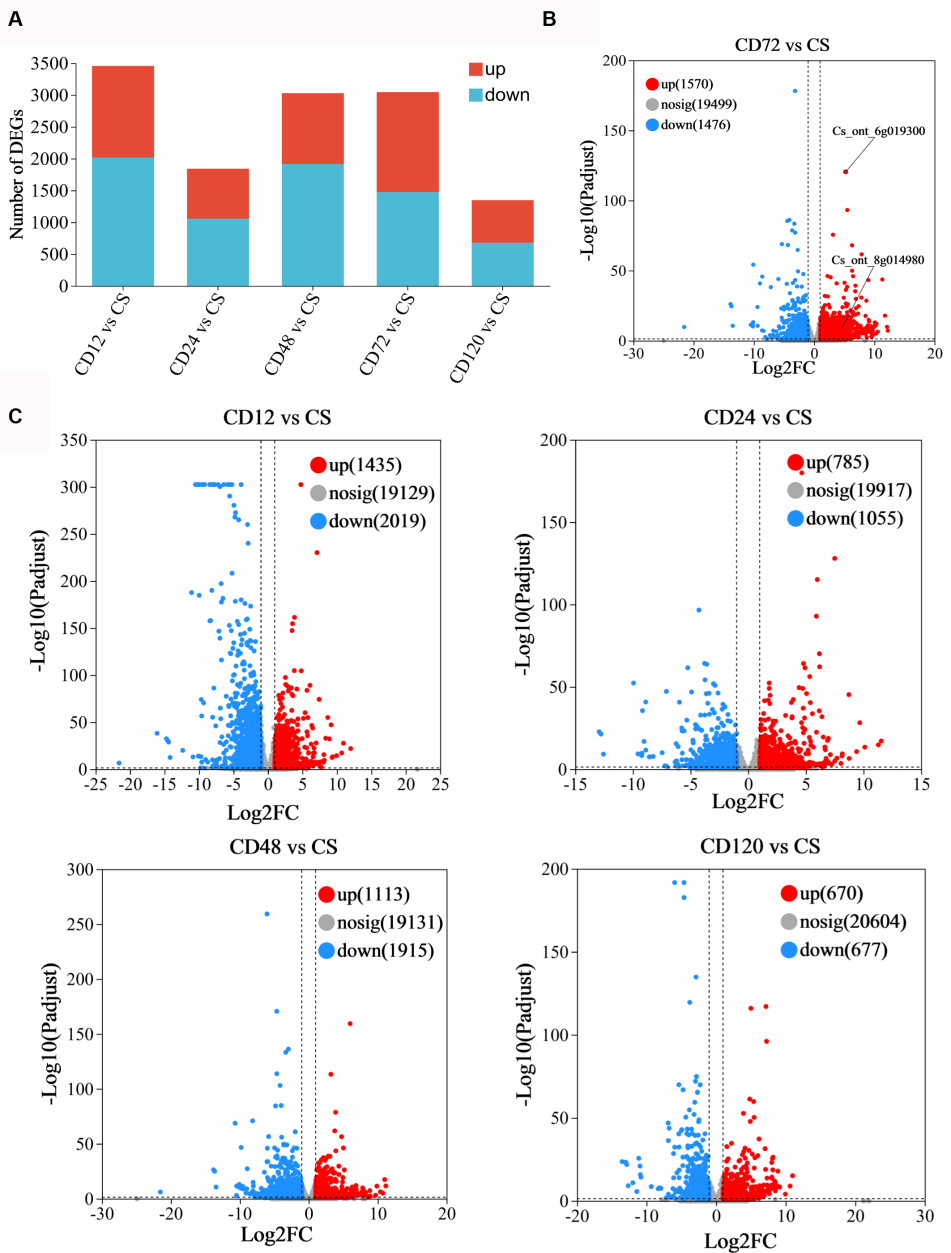
Statistical analysis of differentially expressed genes. **(A)** Statistical map of difference in the expression. Volcano plot of the DEGs in panel **(B)** CD72 vs. Cs (FDR < 0.05 and log_2_FC < −1.0); **(C)** CD12 vs. Cs, CD24 vs. Cs, CD48 vs. Cs, and CD120 vs. Cs as obtained using RNA-Seq. The considerably upregulated (FDR < 0.05 and log_2_FC > 1.0) and downregulated (FDR < 0.05 and log_2_FC < −1.0) DEGs are represented in red and blue, respectively.

Transcriptome analysis was conducted at various time points after *D. citri* infection to study the differential expression profiles of genes of infected and uninfected *C. sinensis* leaves. The RNA-Seq data consisted of the unprocessed reads obtained from five biological replicates of *C. sinensis* leaves at different time points (0, 12, 24, 48, 72, and 120 h) after *D. citri* infection (Table 1). Statistical analysis was conducted on a set of 25,557 expressed genes to assess the differential gene expression in CD12 vs. Cs, CD24 vs. Cs, CD48 vs. Cs, CD72 vs. Cs, and CD120 vs. Cs (Figure 3A). Among them, a total of 1,742 novel genes were identified. In CD12 vs. Cs, CD24 vs. Cs, CD48 vs. Cs, CD72 vs. Cs, and CD120 vs. Cs, 3,454 (41.5% upregulated and 58.5% downregulated), 1,840 (42.7% upregulated and 57.3% downregulated), 3,028 (36.8% upregulated and 63.2% downregulated), 3,046 (51.5% upregulated and 48.5% downregulated), and 1,347 (49.8% upregulated and 50.2% downregulated) DEGs were obtained, respectively (Figure 3A; Supplementary Table S2). All these DEGs met the criteria for significance [−1 > FC >1; *p* < 0.05].

**Table 1:** Mapping results of RNA-Seq reads.

Sample	Total reads	Total mapped	Multiple mapped	Uniquely mapped
CS1	59,452,392	55,275,354(92.97%)	2,176,350(3.66%)	53,099,004(89.31%)
CS2	62,108,354	57,335,380(92.32%)	2,133,512(3.44%)	55,201,868(88.88%)
CS3	57,041,452	52,694,887(92.38%)	1,919,566(3.37%)	50,775,321(89.01%)
CD12_1	219,759,920	204,370,115(93.0%)	7,032,412(3.2%)	197,337,703(89.8%)
CD12_2	264,025,778	244,741,751(92.7%)	9,048,111(3.43%)	235,693,640(89.27%)
CD12_3	269,963,300	249,663,084(92.48%)	8,867,724(3.28%)	240,795,360(89.2%)
CD24_1	98,102,276	91,611,098(93.38%)	3,496,544(3.56%)	88,114,554(89.82%)
CD24_2	105,190,070	98,601,314(93.74%)	3,765,884(3.58%)	94,835,430(90.16%)
CD24_3	89,641,600	83,697,442(93.37%)	3,024,513(3.37%)	80,672,929(89.99%)
CD48_1	163,880,390	151,888,912(92.68%)	6,073,611(3.71%)	145,815,301(88.98%)
CD48_2	183,170,246	169,929,034(92.77%)	6,660,901(3.64%)	163,268,133(89.13%)
CD48_3	177,561,322	165,248,132(93.07%)	6,121,870(3.45%)	159,126,262(89.62%)
CD72_1	182,156,722	168,740,478(92.63%)	6,406,454(3.52%)	162,334,024(89.12%)
CD72_2	190,195,722	175,914,974(92.49%)	6,870,538(3.61%)	169,044,436(88.88%)
CD72_3	184,496,348	170,244,842(92.28%)	6,577,014(3.56%)	163,667,828(88.71%)
CD120_1	96,362,944	90,422,366(93.84%)	3,446,880(3.58%)	86,975,486(90.26%)
CD120_2	84,501,856	78,725,881(93.16%)	2,876,798(3.4%)	75,849,083(89.76%)
CD120_3	97,681,778	91,035,839(93.2%)	3,320,043(3.4%)	87,715,796(89.8%)

The volcano plots were generated to visually represent the differential expression of genes, with the log_2_FC plotted against the negative logarithm (base 10)-transformed FDR values (Figures 3B, 3C). The DEGs exhibiting higher log(10)-transformed FDR values suggested a more pronounced regulation in response to *D. citri* infection. Positive FC values indicate upregulated DEGs with an FDR < 0.05 and log_2_FC > 1.0, whereas negative FC values indicate downregulated DEGs (FDR < 0.05; log_2_FC < −1.0). The majority of upregulated DEGs were identified at 72 h after inoculation (*n* = 1,570; Figure 3B).

### 3.3 GO and KEGG functional enrichment analyses of the DEGs at 72 h after inoculation

**Figure 4:**
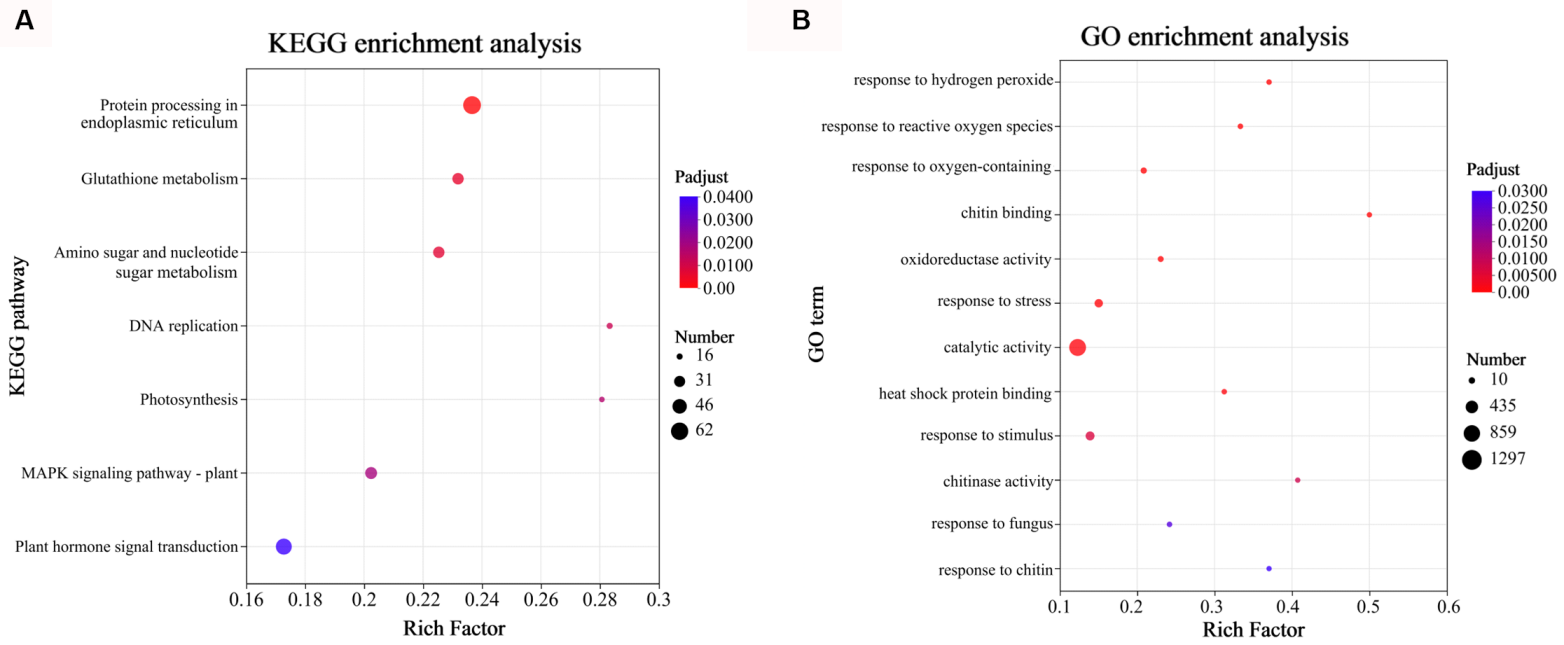
Gene Ontology (GO) and Kyoto Encyclopedia of Genes and Genomes (KEGG) functional enrichment analyses of the DEGs at 72 h after inoculation. **(A)** KEGG functional enrichment analysis was performed on the DEGs in CD72 vs. Cs (Top 7). **(B)** GO enrichment analysis was performed on the DEGs of CD72 vs. Cs associated with active oxygen burst and chitin recognition in *Citrus sinensis* leaves during *Diaporthe citri* infection. Significantly enriched GO terms and KEGG pathways were obtained using Goatools and R script, respectively, using a cutoff of *p* < 0.05.

To assess the enrichment of DEGs in KEGG pathways during *D. citri* infection, KEGG pathway functional enrichment analysis was performed using R script. For the total of 3,046 DEGs in CD72 vs. Cs, 7 metabolic pathways involving 223 genes were identified (*p*-adjust < 0.05; Figure 4A). The KEGG pathway category with the highest number of genes (*n* = 62), and most significant enrichment was “Protein processing in endoplasmic reticulum,” followed by “Glutathione metabolism” and “MAPK signaling pathway-plant.” To better understand the active oxygen burst process in *C. sinensis* leaves after *D. citri* infection, DEGs of CD72 vs. Cs related to active oxygen burst and chitin recognition were functionally enriched and categorized using Goatools software (*p* < 0.05), with Fisher’s exact test (Figure 4B). DEGs of CD72 vs. Cs related to active oxygen burst and chitin recognition were significantly grouped into the biological process [7 GO terms were listed such as response to oxygen-containing compound (GO:1901700), response to ROS (GO:0000302), molecular function (GO:0042542), response to stress (GO:0006950), response to stimulus (GO:0050896), response to fungus (GO:0009620), and response to fungus (GO:0010200)] and molecular function [5 GO terms were listed such as chitin binding (GO:0008061), oxidoreductase activity (GO:0016709), catalytic activity (GO:0003824), heat shock protein binding (GO:0031072), and chitinase activity (GO:0004568)] (Figure 4B).

### 3.4 Clustering analysis and PPI network analysis of specific genes

**Figure 5:**
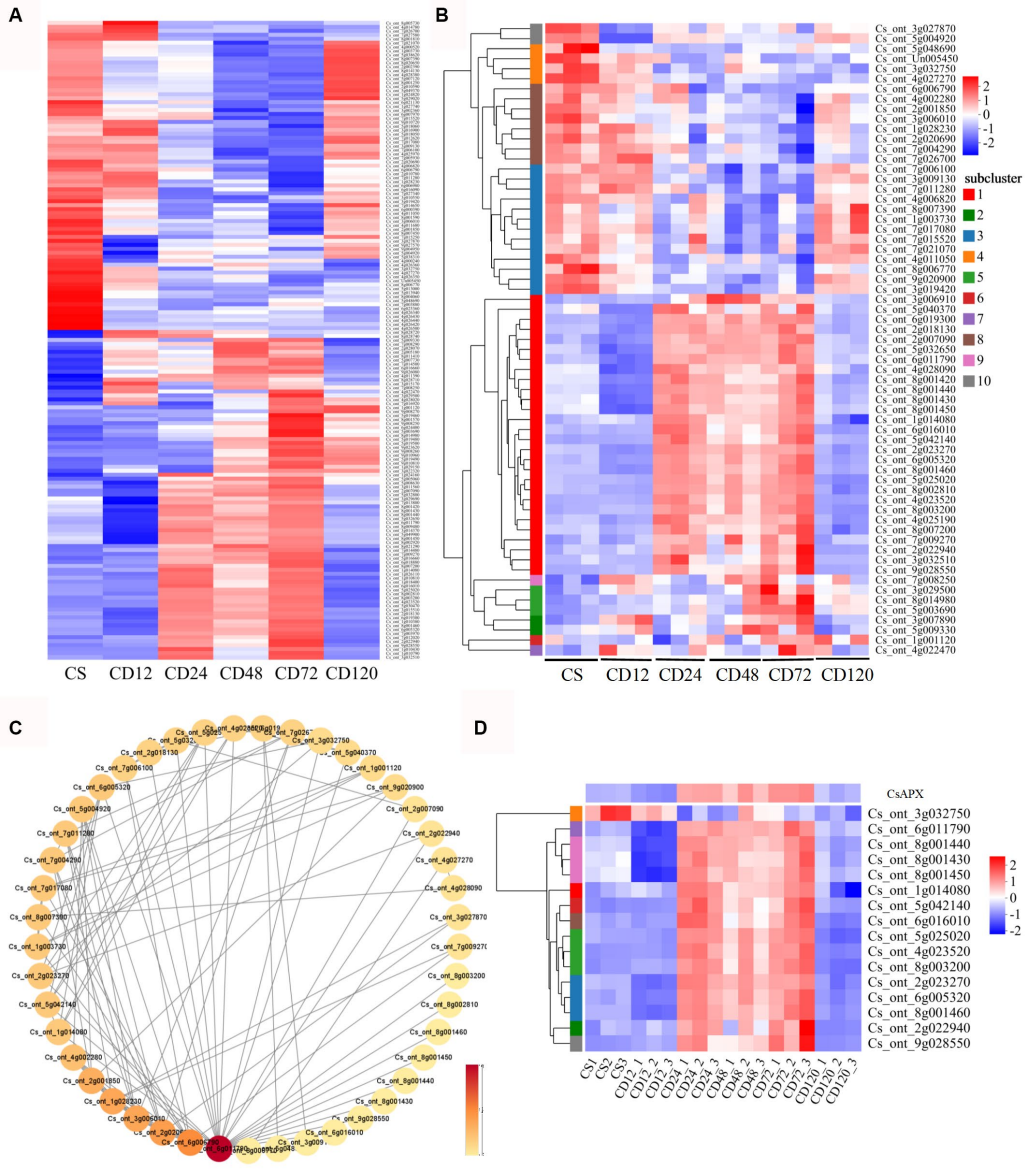
Hierarchical cluster heatmaps and protein–protein interaction (PPI) analysis of specific genes. **(A)** Hierarchical cluster heatmaps were generated for 161 DEGs that were identified in both GO and KEGG analyses of the DEGs of CD72 vs. Cs, at different time points during *D. citri* infection compared with uninfected plants (Cs). Hierarchical cluster heatmaps **(B)** and PPI **(C)** analysis were performed for 63 ROS-related genes identified in both the GO and KEGG analyses of CD72 vs. Cs. The heatmap represents the log_2_fold change (FC). Each node corresponds to a protein, and lines depict interactions between proteins. The color intensity of a node reflects its degree of connectivity. Darker color indicates higher connectivity, which is determined by the number of connections to that particular node. **(D)** The log_2_FC of the HSP genes and *CsAPX* in the PPI network is represented in the heatmap. The values represent the log_2_FC value of the DEGs at the respective time point.

Among the common DEGs identified in the GO and KEGG analyses of DEGs of CD72 vs. Cs, 161 were associated with chitin recognition, redox reactions, or potential redox control (Supplementary Table S3). The expression of 161 DEGs during infection was validated using a heatmap. The results indicated that most genes were upregulated at some stage of *D. citri* infection (Figure 5A).

Furthermore, hierarchical cluster heatmaps (Figure 5B) were plotted for 63 ROS-related genes selected from 6 GO terms. Importantly, oxido-reductases were upregulated in response to the infection after 24, 48, and 72 h (28 genes; Figure 3B; Supplementary Figure S2; Supplementary Table S4), and the results were consistent with those of DAB staining during infection (Figure 2). In addition, among the 63 DEGs specific to *D. citri* infection, 3 were significantly upregulated only in CD72 vs. Cs (Figure 4B; Supplementary Figure S2; Supplementary Table S4). Meanwhile, PPI networks of 63 ROS-related DEGs were constructed to facilitate further identification of genes situated in core positions. The PPI network displayed a total of 45 DEGs with protein interactions (Figure 5C). Overall, 16 HSP genes (*Cs_ont_6g011790*, *Cs_ont_1g014080*, *Cs_ont_2g022940*, *Cs_ont_2g023270*, *Cs_ont_4g023520*, *Cs_ont_5g042140*, *Cs_ont_5g025020*, *Cs_ont_6g016010*, *Cs_ont_6g005320*, *Cs_ont_8g003200*, *Cs_ont_8g001460*, *Cs_ont_8g001450*, *Cs_ont_8g001440*, *Cs_ont_8g001430*, *Cs_ont_9g028550*, and *Cs_ont_3g032750*) were included in these core genes, the majority of which exhibited increased expression compared with the control at 24, 48, and 72 h after inoculation. In addition, a gene related to peroxidase family was identified as *Cs_ont_6g019300* (CsAPX), which exhibited gene expression pattern similar to that of HSP genes (Figure 5D).

### 3.5 Validation of the RNA-Seq analysis

To ensure the credibility and precision of the transcriptome sequencing, 22 DEGs were randomly chosen from different functional categories of DEGs for validation using qRT-PCR at each time point after *D. citri* infection. The *COX* gene served as a reference gene for normalizing the data. Table 2 presents the qRT-PCR results for these 22 genes. The relative expression levels obtained from qRT-PCR were converted to log_2_FC to facilitate direct comparison with RNA-Seq data. Remarkably, the qRT-PCR analysis results were consistent with the results of transcriptome sequencing, thereby confirming the robustness and credibility of the transcriptome sequencing results.

**Table 2:** Comparison of gene expression levels obtained from RNA-Seq and qPCR (*p*<0.05).

Gene ID	Gene name	Sample^a^	Log_2_ Flod change from RNA-Seq	Log_2_ Flod change from qPCR	Gene ID	Gene name	Sample^a^	Log_2_ Flod change from RNA-Seq	Log_2_ Flod change from qPCR
**Oxidative stress related genes**									
Cs_ont_2g034230	*CsPOD*	Cs	0a	0 ± 0.05a	Cs_ont_8g014980	*CsRBOH*	Cs	0a	0 ± 0.24a
		CD12	2.04b	2.95 ± 0.74b			CD12	0.55b	0.75 ± 0.32b
		CD24	–0.1c	0.16 ± 0.39c			CD24	1.83c	2.36 ± 0.68c
		CD48	1.32d	−0.58 ± 0.35d			CD48	2.42d	3.09 ± 1.40d
		CD72	2.1e	2.46 ± 0.06e			CD72	4.58e	3.96 ± 0.74e
		CD120	2.62f	4.9 ± 0.30f			CD120	2.37f	2.82 ± 0.21f
Cs_ont_1g009760	*CsDAO*	Cs	0a	0 ± 0.06a	Cs_ont_6g019300	*CsAPX*	Cs	0a	0 ± 0.15a
		CD12	2.03b	3.41 ± 0.51b			CD12	−1.53b	−1.25 ± 0.44b
		CD24	0.42c	0.4 ± 0.06c			CD24	4.64c	1.78 ± 1.08c
		CD48	1.35d	−2.82 ± 0.35d			CD48	4.36d	4.77 ± 0.30d
		CD72	−0.12e	0.3 ± 0.27e			CD72	5.24e	5.16 ± 0.30e
		CD120	1.10f	5.27 ± 0.39f			CD120	0.11f	0.85 ± 1.10f
Cs_ont_8g006180	*CsSOD1*	Cs	0a	0 ± 0.04a	Cs_ont_6g011790	*CsHSP20*	Cs	0a	0 ± 0.14a
		CD12	−0.53b	1.18 ± 0.16b			CD12	−1.31b	−1.05 ± 0.25b
		CD24	−0.14c	−0.55 ± 0.35c			CD24	1.87c	1.68 ± 0.11c
		CD48	−1.11d	−0.58 ± 0.74d			CD48	1.63d	1.13 ± 0.45d
		CD72	−1.65e	−1.65 ± 0.27e			CD72	2.06e	3.26 ± 0.55e
		CD120	−0.81f	0.74 ± 0.35f			CD120	0.13f	0.14 ± 0.14f
Cs_ont_1g016620	*CsPDXS*	Cs	0a	0 ± 0.02a	Cs_ont_8g007200	*CsCAT*	Cs	0a	0 ± 0.09a
		CD12	−1.64b	−1.08 ± 0.25b			CD12	−1.10b	7.03 ± 0.82b
		CD24	2.15c	1.54 ± 0.23c			CD24	3.79c	12.42 ± 1.74c
		CD48	1.10d	3.50 ± 0.33d			CD48	1.76d	10.34 ± 0.43d
		CD72	2.29e	4.83 ± 0.62e			CD72	4.56e	13.32 ± 1.57e
		CD120	−0.37f	0.08 ± 0.02f			CD120	−1.78f	8.43 ± 0.52f
**Defense response related genes**									
Cs_ont_8g004060	*CsMPK3*	Cs	0a	0 ± 0.02a	Cs_ont_8g027120	*CsPR1*	Cs	0a	0 ± 0.03a
		CD12	−1.91b	0.19 ± 0.35b			CD12	4.21b	4.47 ± 0.68b
		CD24	−2.01c	−2.48 ± 0.74c			CD24	4.94c	5.23 ± 0.39c
		CD48	−2.31c	−2.87 ± 0.35d			CD48	8.08d	2.88 ± 0.11d
		CD72	−2.02d	−1.88 ± 0.51e			CD72	10.02e	8.7 ± 0.39e
		CD120	−2.01e	−0.1 ± 0.30f			CD120	10.62f	10.05 ± 0.35f
Cs_ont_5g048470	*CsHSP90*	Cs	0a	0 ± 0.16a	Cs_ont_9g026670	*CsSGT1*	Cs	0a	0 ± 0.08a
		CD12	−2.48b	−2.26 ± 0.71b			CD12	−0.38b	0.27 ± 0.62a
		CD24	6.17c	6.13 ± 0.26c			CD24	0.80c	0.88 ± 0.35b
		CD48	5.03d	5.53 ± 0.51d			CD48	0.63d	0.88 ± 0.33c
		CD72	6.84e	5.56 ± 0.35e			CD72	1.07d	0.70 ± 0.37d
		CD120	−1.08f	−0.61 ± 0.06f			CD120	0.00e	0.22 ± 0.27e
**Hormone related genes**									
Cs_ont_9g010770	*CsGST*	Cs	0a	0 ± 0.05a	Cs_ont_7g015010	*CsHPT*	Cs	0a	0 ± 0.02a
		CD12	2.76b	4.98 ± 0.70b			CD12	4.19b	0.66 ± 0.11b
		CD24	3.23c	3.50 ± 0.18c			CD24	6.93c	3.77 ± 0.01c
		CD48	2.82d	2.34 ± 0.61d			CD48	9.69d	2.89 ± 0.03d
		CD72	5.54e	7.00 ± 0.79e			CD72	12.23e	7.98 ± 0.03e
		CD120	2.12f	3.75 ± 0.25f			CD120	10.99f	7.26 ± 0.04f
Cs_ont_5g003690	*CsACS1*	Cs	0a	0 ± 0.07a	Cs_ont_8g010200	*CsPR-4A*	Cs	0f	0 ± 0.01f
		CD12	2.23b	3.42 ± 0.72b			CD12	2.59d	5.82 ± 0.03e
		CD24	3.45c	2.36 ± 0.28c			CD24	2.53e	8.58 ± 0.07d
		CD48	3.86d	1.37 ± 0.36d			CD48	4.36b	11.08 ± 0.44b
		CD72	5.68e	5.64 ± 0.66e			CD72	5.72a	11.67 ± 0.04a
		CD120	3.57f	5.40 ± 0.24f			CD120	3.96c	10.59 ± 0.04c
**Transcription factor related genes**									
Cs_ont_5g004320	*CsWRKY46*	Cs	0a	0 ± 0.17a	Cs_ont_3g019860	*CsNOTUM*	Cs	0a	0 ± 0.04a
		CD12	−2.69b	−1.84 ± 0.21c			CD12	−1.31b	−3.83 ± 0.01d
		CD24	−4.04e	−2.41 ± 0.68e			CD24	−2.19d	−5.70 ± 0.01e
		CD48	−4.11f	−2.31 ± 0.67d			CD48	−4.56f	−5.75 ± 0.01f
		CD72	−3.58c	−1.05 ± 0.20b			CD72	−1.91c	−2.39 ± 0.13b
		CD120	−3.92d	−4.48 ± 0.97f			CD120	−2.37e	−2.57 ± 0.01c
Cs_ont_9g025130	*CsERF027*	Cs	0a	0 ± 0.27a	Cs_ont_1g025050	*CsERF109*	Cs	0a	0 ± 0.17a
		CD12	−6.95b	−4.09 ± 0.06f			CD12	−7.39f	−2.06 ± 0.36c
		CD24	−9.03d	−1.01 ± 0.34c			CD24	−6.34d	−3.39 ± 0.25f
		CD48	−8.93c	−2.27 ± 0.46d			CD48	−6.64e	−1.85 ± 0.20b
		CD72	−9.41e	−3.52 ± 0.33e			CD72	−6.27c	−2.21 ± 0.61e
		CD120	−11.97f	−0.43 ± 0.18b			CD120	−5.83b	−2.13 ± 0.78d
**Chitin recognition related genes**									
Cs_ont_8g028710	*CsCHIB1*	Cs	0a	0 ± 0.04a	Cs_ont_8g028740	*CsCHIB2*	Cs	0a	0 ± 0.13a
		CD12	5.52b	5.47 ± 1.23b			CD12	5.31b	5.69 ± 0.35b
		CD24	5.30c	3.78 ± 0.46c			CD24	5.19c	5.37 ± 0.27c
		CD48	5.51d	5.48 ± 0.49d			CD48	3.28d	3.25 ± 0.35d
		CD72	4.21e	4.54 ± 1.31e			CD72	2.17e	1.87 ± 0.44e
		CD120	1.96f	2.36 ± 0.75f			CD120	3.16f	6.43 ± 0.06f

^a^Samples Cs, CD12, CD24, CD48, CD72, and CD120 represent 0, 12, 24, 48, 72, and 120 h after inoculation with *Diaporthe citri.* Different letters in a column denote statistically significant differences (*P*<0.05).

### 3.6 Overexpression of *CsRBOH* activates plant immune responses in *Nicotiana benthamiana*

Transcriptome analysis results indicated that among the hub genes of *C. sinensis* that responded to *D. citri* infection, the NADPH oxidase gene *RBOHD* (*Cs_ont_8g014980*) was significantly associated with ROS burst in the leaves of *C. sinensis* during the infection. Therefore, *CsRBOH* was selected for further investigation.

**Figure 6:**
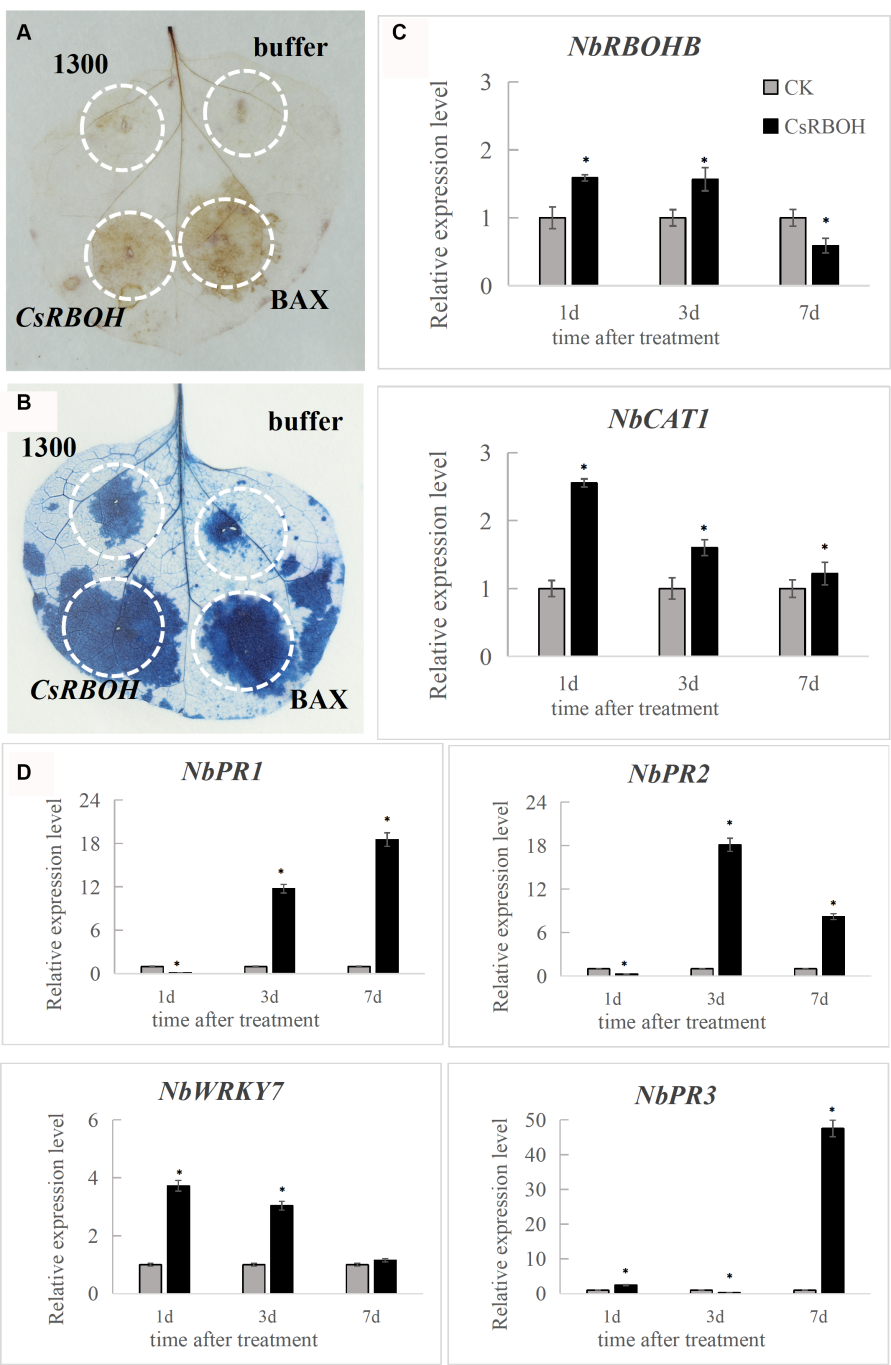
*CsRBOH* activates plant immune responses in *Nicotiana benthamiana*. **(A)** *CsRBOH* could activate ROS accumulation in *N. benthamiana* leaves. *Nicotiana benthamiana* leaves were infected with *A. tumefaciens* expressing *CsRBOH*, BAX (as the positive control), or pCAMBIA-1300 empty vector (as the negative control). DAB staining was used to detect ROS accumulation in *N. benthamiana* leaves 2–3 days postinfiltration (dpi). **(B)** *CsRBOH* could activate the BAX-mediated cell death in *N. benthamiana* leaves. Trypan blue staining was used to detect cell death in *N. benthamiana* leaves 7–8 dpi. **(C)** The expression of two ROS-related genes was analyzed using qRT-PCR after infection with *A. tumefaciens* expressing *CsRBOH*. **(D)** The expression of four defense-related genes was analyzed using qRT-PCR after infection with *A. tumefaciens* expressing *CsRBOH*. The values represent the means (± SE) obtained from three independent experiments. The bars indicate the standard deviations. Asterisks indicate statistical significance (*p ≤ 0.01) using Student’s t-test.

To determine the immune response capacity of *CsRBOH*, *CsRBOH* was inserted into the plant transient expression vector pCAMBIA-1300 plasmid. *Agrobacterium*-mediated transformation system was used to deliver the agrobacteria culture containing constructs into *N. benthamiana* leaves for transient expression. The BAX protein, which belongs to the mouse Bcl-2 family, has demonstrated potent ability to trigger apoptosis in *N. benthamiana* (Lacomme and Santa Cruz, 1999). BAX protein was used as the positive control. DAB staining at 2 dpi indicated that *CsRBOH* (Figure 6A) could induce ROS accumulation in *N. benthamiana* leaves, compared to the negative control (pCAMBIA-1300). At 8 dpi, *CsRBOH* could induce BAX-mediated cell death in *N. benthamiana* leaves (Figure 6B). These findings indicated that the plant defense responses can be triggered by the transient expression of *CsRBOH*. Two genes related to ROS regulation [*NbRBOHB* (related to ROS production) and *NbCAT1* (related to the regulation of H_2_O_2_ homeostasis in plants; Zhang et al., 2015; Chen et al., 2022)] and four defense-related genes [*NbPR1* and *NbPR2* induced by salicylic acid (SA), *NbPR3* induced by jasmonic acid (JA), and *NbWRKY7* related to PAMP-triggered immunity (PTI; Zhang et al., 2010; WANG et al., 2019)] were selected for expression analysis using qRT-PCR after introducing *CsRBOH* in *N. benthamiana* leaves (Figures 6C,D). The expression levels of these genes were significantly increased in *N. benthamiana* leaves after transient expression of *CsRBOH*, suggesting that triggering plant immune responses by *CsRBOH* involves the triggering of SA-, JA-, and/or PAMP-related defense pathways.

## 4 Discussion

Transcriptome analysis of the plants during fungal infection process has recently emerged as an alternative approach to gain insights into the immune response of host against *Diaporthe* infection (Książkiewicz et al., 2021; Mena et al., 2022). However, transcriptome studies involving the direct interaction of *D. citri* with a host are scarce. Recently, Li et al. (2023) employed RNA-Seq to examine the gene expression profile in *Citrus* leaves after infection with *D. citri* at early (3 dpi) and late (14 dpi) stages of infection. They highlighted the variations in transcriptional regulation associated with defense responses (Li et al., 2023). In our study, the transcriptomes of *C. sinensis* leaves at 12 (contact period), 24, 48, 72 (expansion phase), and 120 h (symptomatic period) after inoculation with virulent *D. citri* HJG-1 strain were analyzed and compared with the uninoculated healthy *C. sinensis* leaves.

ROS play pivotal roles in stress perception, integration of diverse stress-responsive signaling networks, and activation of plant defense mechanisms and acclimation (Camejo et al., 2016; Mittler et al., 2022). After pathogenic infection, plants quickly initiate PTI by triggering a burst of ROS and deposition of callose (Galletti et al., 2008; Liu et al., 2020). In our study, H_2_O_2_ levels were increased during the early mid stage of *D. citri* infection. The ROS response of *C. sinensis* leaves was significantly reduced after the appearance of black dot symptoms (Figure 2). Additionally, because the leaves were too young at the time of inoculation, the degree of leaf spread was inconsistent. Therefore, there is a certain chance that the spore suspension will gather toward the concave leaf veins due to the influence of leaf morphology. While sampling, we mainly focused on similar leaves in terms of shape and size, ignoring their curvature status. Figure 2A shows that the stained areas were not evenly distributed, whereas Supplementary Figure S1 shows that the black spots were quite evenly distributed on the leaves. However, this issue does not significantly impact the conclusion that pathogenic infection induces ROS burst in the host. Through ROS burst, relevant immune signals may be activated in *C. sinensis* after *D. citri* infection.

The number of expressed genes (25,557) obtained in this study for *C. sinensis* exceeded the gene count reported in previous transcriptome studies (16,896 genes; Li et al., 2023). This can be attributed to enhanced sequencing depth in this study (Table 1) and the difference in the reference genomes (Li et al., 2023). A significant number of genes were upregulated in CD72 vs. Cs (Figure 3A); thus, the DEGs in CD72 vs. Cs were further analyzed. A joint cluster analysis was conducted by integrating these DEGs with those at other different time points. When comparing gene expression profiles of uninfected and infected *C. sinensis* leaves, several transcripts were observed to be related to ROS. Specifically, the transcripts were linked to protein processing in the endoplasmic reticulum (Zheng et al., 2018) and MAPK signaling pathway—plant (Mittler et al., 2022). Interestingly, GO enrichment analysis of the DEGs at 72 h after inoculation (top 20 are listed) revealed that the DEGs were enriched in eight ROS-related GO categories (Supplementary Figure S3). This is consistent with the results of oxidative burst, indicating that oxidative burst occurs in *C. sinensis* during *D. citri* infection.

Several studies have suggested that ROS are crucial molecules during plant-pathogen interactions (Mittler et al., 2011; Camejo et al., 2016). Importantly, ROS accumulation in cells can have damaging, protective, or signaling effects depending on the sophisticated balance between ROS-generating and -scavenging systems at the appropriate site and time (Camejo et al., 2016). Responses to pathogenic infection after pathogen recognition are commonly triggered by a temporary oxidative burst, which is facilitated by RBOHs or peroxidases located at the apoplast (Torres et al., 2002; Daudi et al., 2012). After the burst, the reduced state of the cytoplasm and callose deposition increase at both cell wall and plasmodesmata. This effectively hinders any potential spread of pathogens (Daudi et al., 2012). The redox-regulated transcriptional response mediated by NPR1 is initiated upon pathogen recognition, leading to enhanced accumulation of ROS (Mittler et al., 2022).

The results of qRT-PCR verification indicated that *Cs_ont_2g034230* (*CsPOD*), *Cs_ont_1g009760* (*CsDAO*), and *Cs_ont_8g014980* (*CsRBOH*; encoding peroxidase 15, oxidoreductase, and respiratory burst oxidase homolog, respectively) were significantly upregulated from 12 h after inoculation. Cs_ont_8g007200 (*CsCAT*) and Cs_ont_1g016620 (*CsPDXS*; encoding catalase) were significantly upregulated from 24 to 72 h after the inoculation. *Cs_ont_8g006180* (*CsSOD*), encoding superoxide dismutase, was significantly downregulated from 24 to 72 h after the inoculation. This indicated that the host does not regulate its disease resistance by activating the SOD gene. *Cs_ont_8g028710* (*CsCHIB1*) and *Cs_ont_8g028740* (*CsCHIB2*; encoding chitinase) and *Cs_ont_8g027120* (*CsPR1*; encoding pathogenesis-related protein 1) were significantly upregulated after the inoculation. Hormone related genes *Cs_ont_9g010770* (*CsGST*) and *Cs_ont_8g010200* (CsPR-4A) encoding abscisic acid, as well as *Cs_ont_7g015010* (*CsHPT*) and *Cs_ont_5g003690* (*CsACS1*) encoding ethylene-related genes, were significantly upregulated after the inoculation. This is consistent with the findings of Li et al. (2023). The transcription-factor-related genes Cs_ont_5g004320 (*CsWRKY46*), Cs_ont_3g019860 (*CsNOTUM*), and Cs_ont_9g025130 (*CsERF027*) and Cs_ont_1g025050 (*CsERF109*) encode WRKY, NAC, and ethylene-responsive transcription factors, respectively. These genes were significantly downregulated throughout the infection process. The results suggested that *D. citri* may activate downstream responses by negatively regulating WRKY, NAC, and ERF transcription factors in *C. sinensis*. In addition, *Cs_ont_6g011790* (*CsHSP20*) and *Cs_ont_5g048470* (*CsHSP90*) encoding heat shock protein were significantly upregulated from 24 to 72 h after the inoculation. These genes were enriched in the KEGG pathway of “Protein processing in endoplasmic reticulum” (map04141), and their association with host disease resistance warrants further experimental validation. It is noteworthy that the expression of *Cs_ont_6g019300* (*CsAPX*), which encodes L-ascorbate peroxidase 2, was significantly upregulated from 24 to 72 h after inoculation, exhibiting more than 4-fold increase (Table 2). Its high expression beginning with the metaphase indicated that it could have functions in the phase of expansion during infection. Currently, the *Citrus* genetic transformation technology is tedious and time-consuming (Conti et al., 2021). This hindered the direct determination of gene functions in this study. To overcome this limitation, the transient expression technique using the binary vector pCAMBIA-1300 and *N. benthamiana* leaves was used in this study to explore the functions of *CsRBOH* (Nie et al., 2015; Yan et al., 2022). In *Agrobacterium* GV3101-P19 inoculation assay, *CsRBOH* could activate both hypersensitive response and basal defense (Figure 6). Respiratory burst oxidase homologs (rbohs) are highly researched plant enzymes that generate ROS (Suzuki et al., 2011). These rbohs, which exist as a multigene family, play crucial roles in various signaling pathways such as plant growth and development, as well as responses to abiotic and biotic stress (Li et al., 2015). Various rbohs across a minimum of 27 plant varieties have been reported (Kaur et al., 2014). The association between rboh genes and host disease resistance has been demonstrated in crops including tomato (Li et al., 2015), soybean (Ranjan et al., 2018), and *Arabidopsis* (Torres et al., 2013; Zhu et al., 2013). *AtrbohD* plays a crucial role in the defense response because it is the primary gene responsible for ROS production, whereas AtrbohF exhibits a relatively minor impact (Chen et al., 2017), suggesting functional redundancy within the plant rboh gene family. Studies have demonstrated the significant involvement of *rbohD* in the defense response of *Citrus* against both *C*Las and *Xanthomonas citri* ssp. *Citri*, with a correlation with ROS (Mafra et al., 2013; Mei et al., 2019). In contrast to the findings of Mafra et al. (2013), our study revealed significant upregulation of *CsRBOH* in both RNA-Seq and qRT-PCR analyses after pathogenic infection (Table 2). Importantly, Gong et al. (2023) recently reported an efficient and stable technique for genetic transformation of roots in woody plants, which is important for obtaining *Citrus* transgenic plants. In our future studies, we will consider applying this technique for gene function validation to enhance the persuasiveness of the validation results (Ramasamy et al., 2023).

In addition, *CsRBOH* overexpression regulating the intracellular ROS level led to a notable upregulation of four defense-related genes (*NbPR1*, *NbPR2*, *NbPR3*, and *NbWRKY7*) in *N. benthamiana* leaves (Figures 6C,D). Plants rely on PTI and effector-induced immunity to recognize intruding pathogens and subsequently initiate defense responses (Chang et al., 2022). Hence, our findings suggested that *CsRBOH* may defend fungal invasion by activating plant defense responses. *CsRBOH* affected the expression of the defense marker genes *NbPR1*, *NbPR2*, *NbPR3*, and *NbWRKY7*; therefore, the regulatory elements located upstream of these genes may be affected during the activation process of these genes. To enhance the understanding of the fundamental mechanism underlying cell death activation by *CsRBOH*, future studies should focus on elucidating the process of protein activation within host cells and its recognition by cytoplasmic receptors to trigger cell death. Kimura et al. (2020) reported that in *A. thaliana*, *CRK2* and *RBOHD* form a preactivation complex, leading to the phosphorylation of the C-terminus of *RBOHD in vivo* for regulating ROS production. Future studies should verify whether the orthologous genes reported in this study (*CsRBOH*) function through the same pathway.

## Data availability statement

The datasets utilized in this research are available at the NCBI SRA database under accession number PRJNA1111846 (https://www.ncbi.nlm.nih.gov/bioproject/PRJNA1111846).

## Author contributions

TL: Conceptualization, Data curation, Formal analysis, Methodology, Software, Visualization, Writing – original draft, Writing – review & editing. ZZ: Conceptualization, Investigation, Methodology, Resources, Writing – review & editing. CL: Investigation, Methodology, Software, Validation, Writing – original draft. HL: Resources, Writing – review & editing. JT: Funding acquisition, Investigation, Project administration, Resources, Writing – review & editing. XS: Investigation, Resources, Supervision, Writing – review & editing. DL: Formal analysis, Investigation, Methodology, Resources, Writing – review & editing. QZ: Funding acquisition, Investigation, Methodology, Resources, Writing – review & editing. JL: Data curation, Funding acquisition, Software, Writing – review & editing. YX: Project administration, Resources, Writing – review & editing. NS: Funding acquisition, Methodology, Project administration, Resources, Writing – review & editing. TY: Conceptualization, Funding acquisition, Project administration, Resources, Writing – review & editing.

## Footnotes

^1^ https://scipy.org/install/

## Supplementary material

SUPPLEMENTARY FIGURE S1 Symptoms of black spots on leaves after inoculation with *Diaporthe citri*.

SUPPLEMENTARY FIGURE S2 Normalized expression of subcluster 1 and subcluster 5 in Hierarchical cluster heatmaps analysis for 63 ROS genes (28 genes).

SUPPLEMENTARY FIGURE S3 GO enrichment analysis of DEGs in the 72 h after inoculation (top 20 are listed). The abscissa label represents the rich factor of pathways. The colors of the dots represent the *p*-values of enrichment, and the size of the dots represents the number of enriched genes.
