# Supplementary figures and images for: Elucidation of mechanisms underlying active oxygen burst in Citrus sinensis after Diaporthe citri infection using transcriptome analysis
Source: Front Microbiol. 2024 Aug 29;15:1425441. doi: 10.3389/fmicb.2024.1425441 (PMC11390498; doi:10.3389/fmicb.2024.1425441)

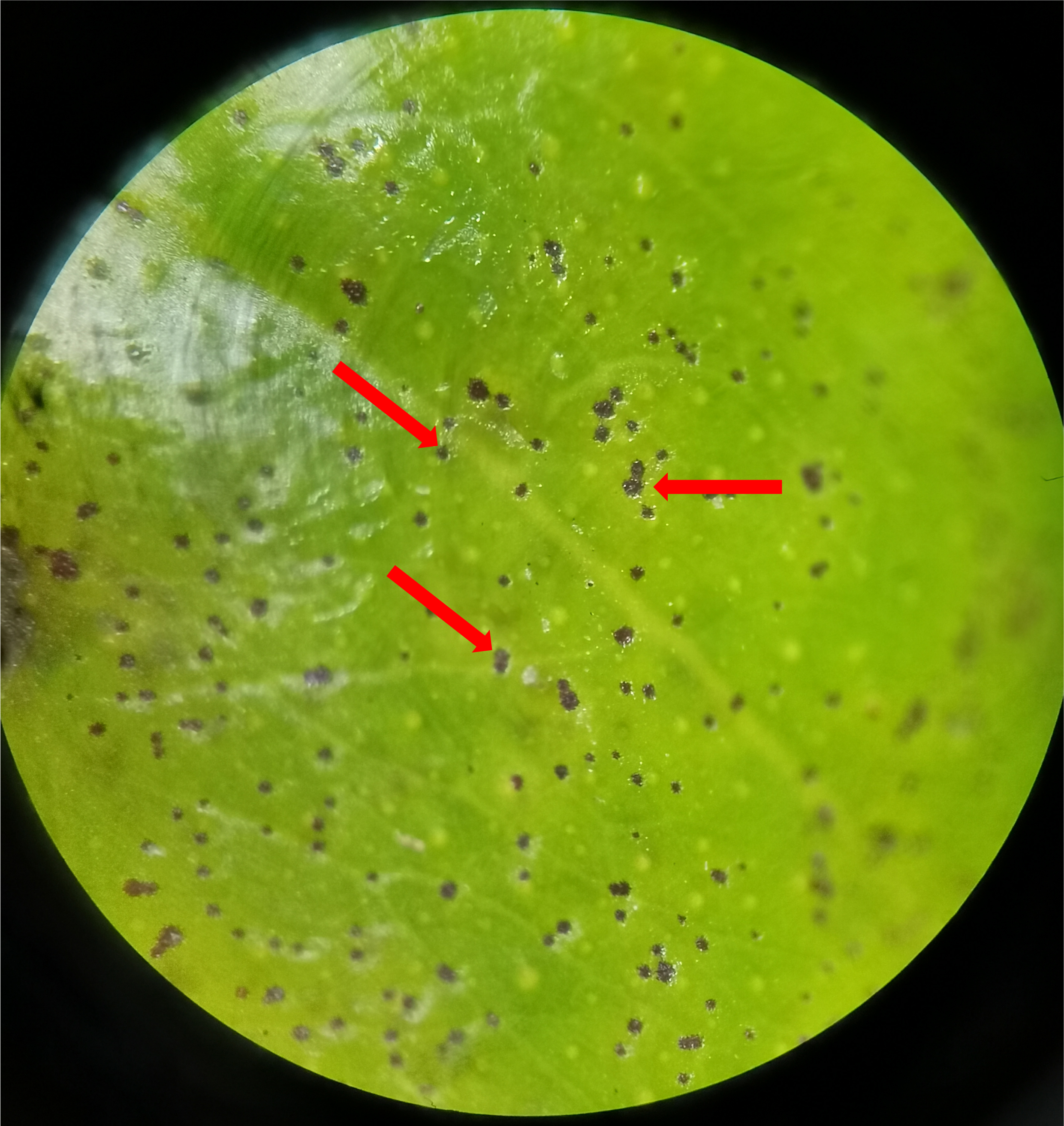

Supplement: SUPPLEMENTARY FIGURE S1 — Symptoms of black spots on leaves after inoculation with Diaporthe citri. [file Image_1.TIF]

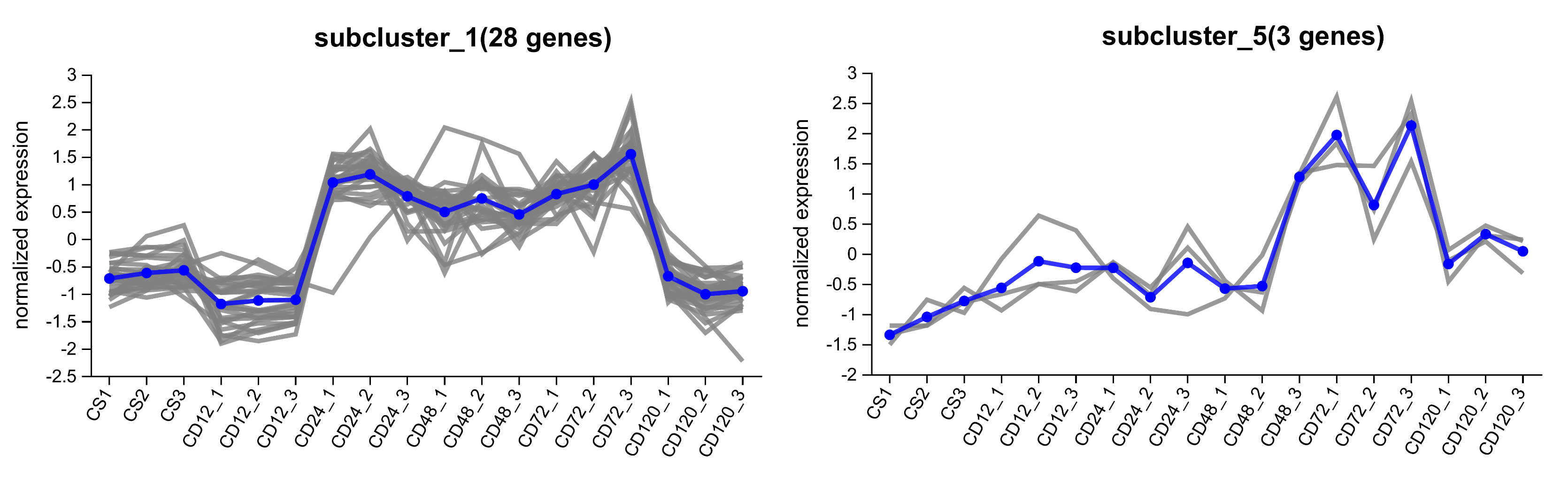

Supplement: SUPPLEMENTARY FIGURE S2 — Normalized expression of subcluster 1 and subcluster 5 in Hierarchical cluster heatmaps analysis for 63 ROS genes (28 genes). [file Image_2.TIF]

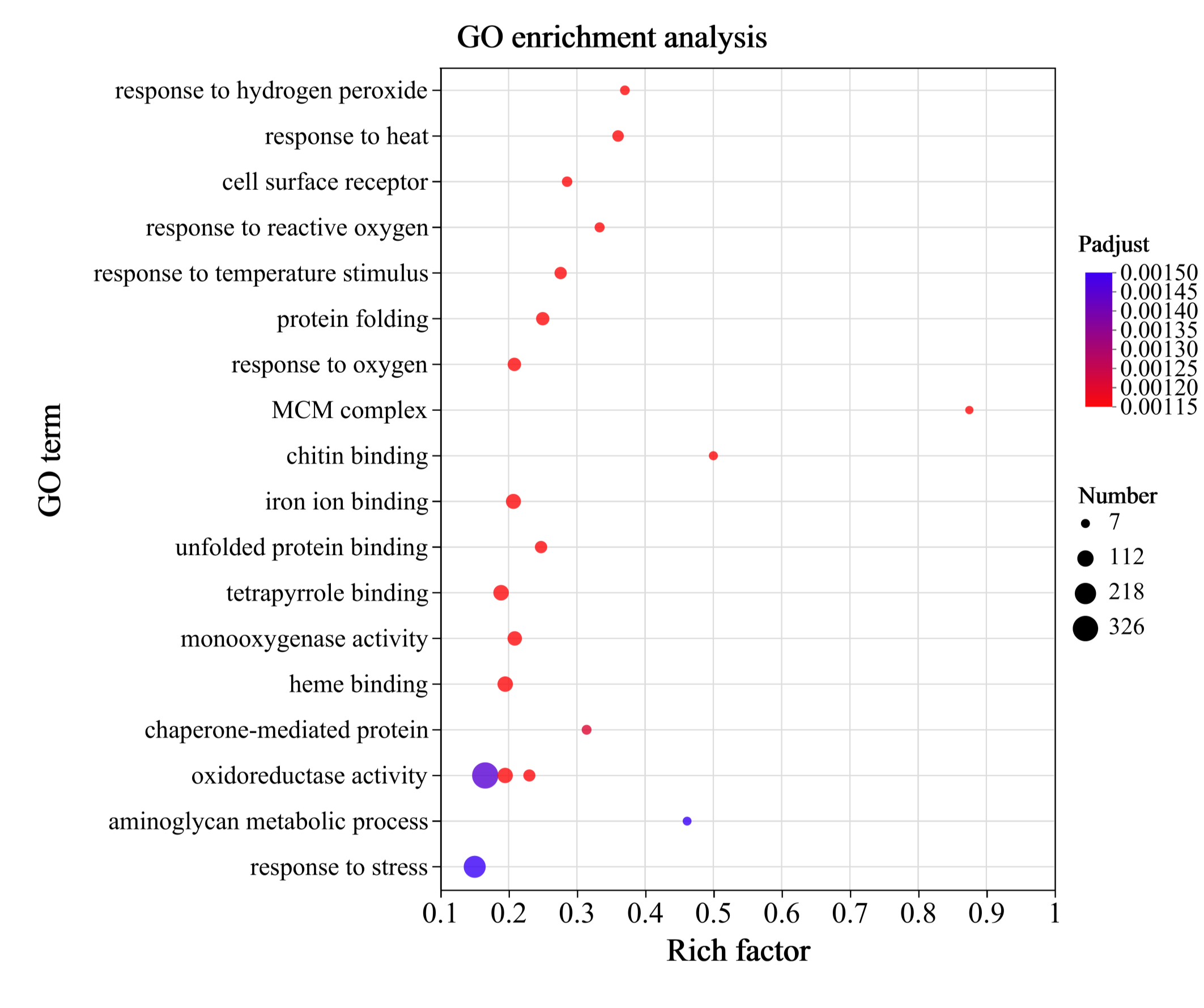

Supplement: SUPPLEMENTARY FIGURE S3 — GO enrichment analysis of DEGs in the 72 h after inoculation (top 20 are listed). The abscissa label represents the rich factor of pathways. The colors of the dots represent the p-values of enrichment, and the size of the dots represents the number of enriched genes. [file Image_3.TIF]
