# Supplementary material for: Elucidation of mechanisms underlying active oxygen burst in Citrus sinensis after Diaporthe citri infection using transcriptome analysis
Source: Front Microbiol. 2024 Aug 29;15:1425441. doi: 10.3389/fmicb.2024.1425441 (PMC11390498; doi:10.3389/fmicb.2024.1425441)
Supplement: Supplementary file 4 [file Table_2.DOCX]

| **Table S1.** Primers used in this study | | |
| --- | --- | --- |
| **Primer name** | **Primer Sequence (5'-3')** | **Reference** |
| qRT-PCR validation of DEGs | | |
| CsCOX-F | GTATGCCACGTCGCATTCCAGA | Song et al., 2021 |
| CsCOX-R | GCCAAAACTGCTAAGGGCATTC |  |
| CsMPK3-F | GCTGCGGAACTCATGGAC | Present work |
| CsMPK3-R | GGGCTCGGTACCATCTTGTGA |  |
| CSPR1-F | CCCCAAGACTATGTCAATGCT | Present work |
| CSPR1-R | TGCAGTCGCCTTTACGTTG |  |
| CsSOD1-F | ACGGGTTCCACCTACACGAA | Present work |
| CsSOD1-R | GTCGCACTTCATCTTTAGGAGC |  |
| CsPOD-F | GGGCAATTTGAAACCACTGACA | Present work |
| CsPOD-R | TGCGATATTGCTGTTTCCGTTG |  |
| CsDAO-F | TGCCTCCAATTCCACTTGAGC | Present work |
| CsDAO-R | GCGTGTACCGTTTATGCACCT |  |
| CsHSP90-F | GGGCTTAACCGACAAGAGCA | Present work |
| CsHSP90-R | GGCCTCCATAAACTCCTTCGT |  |
| CsSGT1-F | TGCGAACCGAGCAATTGAGT | Present work |
| CsSGT1-R | GTGCCACCTTAGCCGTTT |  |
| CsWRKY46-F | TTCCAAGAGGGTATTACAGGTG | Present work |
| CsWRKY46-R | TGGCTACACTTATGTCTCCCTC |  |
| CsRBOH-F | ACGGCCTTCTTCATTGCT | Present work |
| CsRBOH-R | CGTCGGAAATCTGGTCCCAA |  |
| CsHSP20-F | TGGCAAACCGTTCAAGCAAG | Present work |
| CsHSP20-R | CACCGCATAACTCAACACCA |  |
| CsCHIB1-F | CGGGATACGGTCTAACCACA | Present work |
| CsCHIB1-R | CAGCGTCATTCCCAACGTAG |  |
| CsCHIB2-F | GGGCAAGATCATCTCAAGGGA | Present work |
| CsCHIB2-R | GGCAGCAATTTCTCTTTTACGC |  |
| CsNOTUM-F | GATTGTTCTAAGCGAAGGGAT | Present work |
| CsNOTUM-R | TGCCCCATCGCAGTATCT |  |
| CsAPX-F | GCAGCAGTTTCCTATCTTGTCC | Present work |
| CsAPX-R | TCCGGTAACTTCAACGGCAAC |  |
| CspdxS-F | AGTGAGTGATGCCGAAGAAG | Present work |
| CspdxS-R | ACTGCTTGCTTGATTTCCTTA |  |
| CsCAT-F | TTGCTTTCCCCTCACTTTC | Present work |
| CsCAT-R | GGGTTTCCTTCCAGTCCAT |  |
| CsERF109-F | TTTCTTCAAATGGTAAAGGGAT | Present work |
| CsERF109-R | CGTTACAGCATCTGGGAGG |  |
| CsERF027-F | CAAGGGGTTCTGGGTCTG | Present work |
| CsERF027-R | AACGAACTCTTGCCCCAT |  |
| CsPR-4A-F | TCTGCCCAAAGTGCTTCTAA | Present work |
| CsPR-4A-R | CCGTCAAGTCCCATCCAA |  |
| CsGST-F | GCATTTGGTTCTATTGTTCATT | Present work |
| CsGST-R | AAAAGACCAACAGTTCATCCC |  |
| CsACS1-F | AAGAAGCCAGCATCAACATC | Present work |
| CsACS1-R | GCATAAATCTCGTCGCATACA |  |
| CsHPT-F | TTATTGAAGCCCTTGGTGC | Present work |
| CsHPT-R | GGCTTGTTGACCTTGTCTATTT |  |
| RT-qPCR analysis of defense related genes in tobacco | | |
| qNbActin-97-F | GCAGGAATCCACGAGACTACA | Qin, 2023 |
| qNbActin-97-R | AACCTCCAATCCAGACACTGT |  |
| NbRBOHB-F | GTTTGCCAGCCACCACCTAAT | Chen et al., 2022 |
| NbRBOHB-R | AAGAGCAGAACGAGCATCACC |  |
| NbCAT1F | CATACGTTTGCCTGGTTCTTC | Zhanget al.,2015 |
| NbCAT1R | CGACCCTAATAGCCTCTTCCT |  |
| RT-NbPR1F | TGGTCAATACGGCGAAAAC | Qin, 2023 |
| RT-NbPR1R | GAACCCTAGCACATCC |  |
| RT-NbPR2F | GCACGACATAACCTTCCACTCTTAG | Qin, 2023 |
| RT-NbPR2R | ACCCTGCTGAATTTGTTCCTTG |  |
| RT-NbPR3F | GGCATTGGTTCTATTGT | Qin, 2023 |
| RT-NbPR3R | AATTTCTTTCCTACGGGCAGTATCATC |  |
| qRT-NbWRKY7-F | CACAAGGGTACAAACAACACAG | Qin, 2023 |
| qRT-NbWRKY7-R | GGTTGCATTTGGTTCATGTAAG |  |
