# Supplementary material for: Elucidation of mechanisms underlying active oxygen burst in Citrus sinensis after Diaporthe citri infection using transcriptome analysis
Source: Front Microbiol. 2024 Aug 29;15:1425441. doi: 10.3389/fmicb.2024.1425441 (PMC11390498; doi:10.3389/fmicb.2024.1425441)
Supplement: Supplementary file 8 [file Table_6.DOCX]

**Independent Review Report, Reviewer 2**

**1.**Revise and enhance the introduction section to provide a more comprehensive and clear overview, incorporating relevant information and discussions to improve the article's quality and readability.

Comments：Thank you for your suggestions. We have refined and revised the introductory section.

**2.**Focus less on introducing advanced transcriptome research and gene expression analysis technologies, and instead emphasize the differences in disease resistance/susceptibility among different citrus varieties, crucial for explaining the choice of sweet orange as the experimental subject.

Comments：This part has been added in the third paragraph of the introduction.

**3.**The article's description of strong immune responses induced by ROS in sweet oranges after pathogen infection contradicts the portrayal of sweet oranges as susceptible to *D. citri*, leading to reader confusion. It is essential to introduce disease and host resistance in the article.

Comments：This part has been added to the four paragraphs of the introduction. *D. citri* is a weak fungus that infests the host, causing the host to produce a reaction in order to inhibit the growth of the pathogen. This reaction leads to the formation of black spot symptoms on citrus branches, leaves, and fruits. The pathogen induces ROS and other reactions within the host, resulting in disease symptoms; however, further research is needed to understand the mechanism behind this induction.

**4.**Is the chosen range of |log2 fold change (FC)| ≥ 1 reasonable for defining DEGs? Typically, a suggested lower threshold value (e.g., |log2 fold change (FC)| ≥ 1.58) is used.

Comments：Thank you for your suggestion. But the threshold value for defining DEGs was chosen as |log2 fold change (FC)| ≥ 1 in order to have a more comprehensive understanding of the hosts in response to *D.citri* infestation by referring to the literature of other researchers.

**5.**Some genes selected in Table 2 and the corresponding part of Figure 6 do not exhibit significantly different expression levels but show a similar expression pattern. It is recommended to include more significant DEGs.

Comments：Thank you very much for your suggestion. We have closely examined the differential expression levels of the genes, and based on the results of the significant difference analysis, we observed that most of the selected genes exhibit differential expression. However, these patterns of differential expression vary at different times of infestation. It is possible that our methods of gene expression analysis may have led to a misunderstanding on your part. Therefore, in accordance with your suggestions, we have included additional DEGs in the manuscript Table 2, categorized and analyzed according to their respective differences. At the same time, we have deleted Figure 6, considering that its content is duplicated in Table 2.

**6.**The corresponding group for 72 h is missing in Figure 1.

Comments：It was confirmed that there is a corresponding group for 72 h in Figure 1, located in the middle of the bottom row of Figure 1, but there is only one field of view, so it may have been missed.

**7.**The absence of significant leaf vein structures in the 120 h leaves in Figure 2 may be due to differences in the shooting angle. Additionally, are the black spots shown disease phenotypes or caused by other factors?

Comments：Firstly, the leaves in Figure 2-120 h are inoculated when they are young and their leaf veins themselves are not fully developed. As a result, the 120 h leaves have been severely affected by D.citri, which has a certain impact on the observation of leaf vein structures, thus making it difficult to observe obvious leaf vein structures. Secondly, the black spots in the figure indeed represent disease phenotypes, which can be observed on unstained and decolorized leaves in Figure S1. Both figures depict pictures of the same infestation stage.

**8.**Figures 7A and 7B may be mirror images, but the positional labels are completely opposite.

Comments：We apologize for the confusion, but we can assure you that Figs. 7A and 7B are two completely different images of two different *N. benthamiana* leaves, as you can see from the veins. The *N. benthamiana* leaves shown in the figures were placed with the leaf surface facing upwards and are the results of the DAB staining and Trypan blue staining tests, respectively.

**9.**Should "RBOH" in the figures be corrected to "CsRBOH"? Considering the extensive research on citrus RBOH related to respiratory burst, verifying gene function through transient transformation in tobacco is not recommended. The availability of rapid transformation materials in citrus rootstock genetic transformation can provide more convincing verification data.

Comments：Thank you very much for pointing out this important issue. We agree with your comments that rapid transformation materials in citrus rootstock genetic transformation can provide more convincing verification data. Your suggestion provides a direction for our next research. Unfortunately, due to the limited time and existing technologies, we did not supplement experimental validation. Despite the inability to make up the experiments, we still believe that the tobacco transient transformation in this paper is an important contribution to the study. To this end, we provide a more in-depth analysis of the theory and additional literature support, and we also analyze and prospects this limitation in the discussion section.

Comments：It has been modified.

**Independent Review Report, Reviewer 3**

**1、**The inoculation assay did not include mock inoculations as controls at 12h, 24h, 48h, 72h, and 120h. Therefore, it is difficult to determine if the stained areas by DAB are due to stress or infection by D. citri. Additionally, Figure 2A shows the stained areas were not evenly distributed, whereas Figure S1 showed the black spots were quite evenly distributed on the leaves.

Comments：During the inoculation test, we have done the water inoculation control including 12 h, 24 h, 48 h, 72 h and 120 h, and we can see from the results that there is a clear difference from the group infected with *D. citri*, and it has been modified in Figure 2. Additionally, because the leaves were too young at the time of inoculation, the degree of leaf spread was inconsistent. Therefore, there is a certain chance that the spore suspension will gather towards the concave leaf veins due to the influence of leaf morphology. While sampling, we mainly focus on consistent leaves in terms of shape and size, ignoring their curvature status. All these factors can affect the distribution of pathogenic bacteria and result in differential phenotypes as you proposed. However, this issue does not significantly impact the conclusion that pathogen infestation induces a burst of ROS in the host. We will address this phenomenon further in discussion.

**2、**Figure 1, it is not clear or is difficult to see what the arrows are pointing to in the image.

Comments：The unclear image in Figure 1 has been replaced with a clearer one.

**3、**The RNA-seq sequence data should be accessible through NCBI under the BioProject ID.

Comments：The RNA-seq sequence data has been uploaded to the NCBI SRA database，SRA accession PRJNA1111846. The SRA records will be accessible with the following link after the indicated release date: <https://www.ncbi.nlm.nih.gov/sra/PRJNA1111846>.

**4、**Table 1 is difficult to understand. Total mapped reads or uniquely mapped reads should not be higher than total reads.

Comments：Thank you for your reminder. After checking, we found that some of the data in Table 1 was incorrectly filled in and has been corrected.

**5、**In Table 2, what was the time point used for the gene expression and RNA-seq?

Comments：The time points used for gene expression and RNA-seq have been described in the notes to Table 2.

**6、**Line 158: What is the reference genome? The authors should indicate the source of citation.

Comments：It has been modified.

**7、**Line 167: The authors need to provide source of citations for the software Goatools and Python SciPy.

Comments：It has been modified.

**8、**Lines 229-234: The authors need to provide all identified DEGs at different time points as a table in the Additional files.

Comments：Added in Additional file 3 of the Supplementary material, with corresponding changes in the manuscript.

**9、**Line 332: The authors need to show and discuss results from all time points, not just 72 hours, based on the number of up-regulated genes. Down-regulated genes also play important roles in the defense response and should not be ignored.

Comments：Thank you very much for your suggestion. It needs to be clarified here that our manuscript mainly analyzes the production of large amounts of ROS induced by *D. citri* in sweet oranges. In the GO and KEGG analyses performed for 72 hours, we combined all differential genes, **including up-regulated and down-regulated genes**, for in-depth analysis. At the same time, we screened key differential genes **at all time points** for gene expression pattern analysis.

**10、**There are inconsistencies in the Additional files. Additional file 5 - Table S3 is identical to the Word document file named Table 4.

Comments：I apologize very much for the duplicate submission of this section, I have corrected it in the Additional files. The order of the supplementary material has also been adjusted according to the order in which it appears in the manuscript.

**11、**The manuscript needs English editing. For example, line 107, ‘Juvenile (1-year-old) C. sinensis (sweet orange) plants’ should be changed to ‘One-year-old sweet orange cultivar plants are known to be susceptible...’. Line 158, change ‘genomein’ to ‘genome in’.

Comments：It has been modified and checked.

**Independent Review Report, Reviewer 4**

**1.**The method for DAB staining is described, but the specificity of this assay for detecting different types of ROS is not mentioned. It is known that DAB primarily detects hydrogen peroxide. It is recommended that hydrogen peroxide and superoxide anions be detected separately.

Comments：We have already added the specificity of the DAB staining method in detecting different types of ROS to the manuscript. The DAB staining method can locate H_2_O_2_, which is the main form of ROS burst in sweet orange leaves. By utilizing the DAB staining method, we clearly demonstrated that *D.citri* infestation can activate the host's ROS burst, while also referencing the research conducted by Liu et al., 2020, and Wang, 2023.

**2.**Some methodology could be clarified further. For example, the steps involved in the transient expression analysis could be detailed to ensure repetition.

Comments：Details and references have been added in Materials and Methods.

**3.**Some references are relatively dated. Updating these references to include more recent studies (post-2020) could provide a more current context to the study and demonstrate the manuscript's relevance to ongoing research in the field.

Comments：The references have been updated.

**4.**Ensure uniformity in figure font sizes; for example, the fonts in Figures 4, 5, and 6 are too small. Additionally, consider enhancing the aesthetic presentation of these figures.

Comments：The figures have been optimized as much as possible based on the suggestions.

**5.** Text formatting within the manuscript should be consistent. For instance, the formatting of (A), (B), and (C) from lines 412 to 433 varies between bold and non-bold, which should be uniform.

Comments：It has been modified and checked.
